# The pro-apoptotic K-Ras 4A proto-oncoprotein does not affect tumorigenesis in the *Apc*^Min/+ ^mouse small intestine

**DOI:** 10.1186/1471-230X-8-24

**Published:** 2008-06-13

**Authors:** Charles E Patek, Mark J Arends, Lorraine Rose, Feijun Luo, Marion Walker, Paul S Devenney, Rachel L Berry, Nicola J Lawrence, Rachel A Ridgway, Owen J Sansom, Martin L Hooper

**Affiliations:** 1 Sir Alastair Currie Cancer Research UK Laboratories, Molecular Medicine Centre, The University of Edinburgh, Western General Hospital, Crewe Road, Edinburgh, EH4 2XU, UK; 2 Department of Pathology, The University of Cambridge, Addenbrooke's Hospital, Hills Road, Cambridge, CB2 2QQ, UK; 3 The Beatson Institute for Cancer Research, Garscube Estate, Switchback Road, Glasgow, G61 1BD, UK; **Current Addresses:**; 4 Comparative and Developmental Genetics, MRC Human Genetics Unit, Western General Hospital, Crewe Road, Edinburgh, EH4 2XU, UK; 5 Division of Oncology, The University of Edinburgh, Western General Hospital, Crewe Road, Edinburgh, ,EH4 2XU, UK; 6 Rheumatic Diseases Unit, Molecular Medicine Centre, The University of Edinburgh, Western General Hospital, Crewe Road, Edinburgh, EH4 2XU, UK; 7 Department of Genetics, Erasmus University Medical Centre, Dr. Molewaterplein, Rotterdam, 3015 GE, The Netherlands

## Abstract

**Background:**

Alterations in gene splicing occur in human sporadic colorectal cancer (CRC) and may contribute to tumour progression. The K-*ras *proto-oncogene encodes two splice variants, K-*ras *4A and 4B, and K-*ras *activating mutations which jointly affect both isoforms are prevalent in CRC. Past studies have established that splicing of both the K-*ras *oncogene and proto-oncogene is altered in CRC in favour of K-*ras *4B. The present study addressed whether the K-Ras 4A proto-oncoprotein can suppress tumour development in the *absence *of its oncogenic allele, utilising the *Apc*^Min/+ ^(*Min*) mouse that spontaneously develops intestinal tumours that do not harbour K-*ras *activating mutations, and the K-*ras*^tmΔ4A/tmΔ4A ^mouse that can express the K-*ras *4B splice variant only. By this means tumorigenesis in the small intestine was compared between *Apc*^Min/+^, K-*ras*^+/+ ^and *Apc*^Min/+^, K-*ras*^tmΔ4A/tmΔ4A ^mice that can, and cannot, express the K-*ras *4A proto-oncoprotein respectively.

**Methods:**

The relative levels of expression of the K-*ras *splice variants in normal small intestine and small intestinal tumours were quantified by real-time RT-qPCR analysis. Inbred (C57BL/6) *Apc*^Min/+^, K-*ras*^+/+ ^and *Apc*^Min/+^, K-*ras*^tmΔ4A/tmΔ4A ^mice were generated and the genotypes confirmed by PCR analysis. Survival of stocks was compared by the Mantel-Haenszel test, and tumour number and area compared by Student's *t*-test in outwardly healthy mice at approximately 106 and 152 days of age. DNA sequencing of codons 12, 13 and 61 was performed to confirm the intestinal tumours did not harbour a K-*ras *activating mutation.

**Results:**

The K-*ras *4A transcript accounted for about 50% of K-*ras *expressed in the small intestine of both wild-type and *Min *mice. Tumours in the small intestine of *Min *mice showed increased levels of K-*ras *4B transcript expression, but no appreciable change in K-*ras *4A transcript levels. No K-*ras *activating mutations were detected in 27 intestinal tumours derived from *Min *and compound mutant *Min *mice. K-Ras 4A deficiency did not affect mouse survival, or tumour number, size or histopathology.

**Conclusion:**

The K-Ras 4A proto-oncoprotein does not exhibit tumour suppressor activity in the small intestine, even though the K-*ras *4A/4B ratio is reduced in adenomas lacking K-*ras *activating mutations.

## Background

The development of sporadic colorectal cancer (CRC) involves genetic and epigenetic changes, including allelic losses in specific chromosomal arms, mutations of oncogenes, tumour suppressor genes and mismatch repair genes, micro-satellite instability, and methylation changes in gene promoters [reviewed [1]]. Additionally, CRC is frequently associated with altered splicing of tumour suppressor genes, and genes encoding enzymes, growth factors, cytoskeletal and cell adhesion proteins, hormone and growth factor receptors, and transcription factors [see [2-5]]. Importantly, since different splice variants of a given gene can have different or even antagonistic effects on diverse cellular functions, including apoptosis, proliferation, differentiation, angiogenesis and cell motility, a change in the splice variant ratio may actively contribute to tumour progression. Indeed, positive selection for splice variants that encode isoforms with a selective advantage in tumour progression is of potential diagnostic value and could provide therapeutic targets.

Ras proteins are low molecular weight (~21 kD) GTPases which cycle between the GDP-bound (inactive) and the GTP-bound (active) state at the plasma membrane, and thereby regulate cell growth, apoptosis, motility and differentiation. K-*ras *activating point mutations occur in about 50% of human sporadic CRC cases and act by stabilising the active GTP-bound configuration, and so promote cellular transformation by constitutive activation of downstream effector pathways, including Raf kinases, phosphatidylinositol 3-kinases (PI3-K), and RalGDS family members [reviewed [6]]. K-*ras *activating mutations play a key role in tumour progression and metastasis in CRC by regulating angiogenesis and protease expression, and cell polarity, adhesion and motility [see [7-9]]. The K-*ras *gene encodes two splice variants, K-*ras *4A and 4B, and activating mutations that usually arise at codons 12, 13 or 61, jointly affect both isoforms [reviewed [6]]. Importantly, since K-Ras oncoproteins differentially promote transformation, cell migration, and anchorage-independent growth, they most probably act in a cooperative manner to drive neoplastic progression [10]. The ratio of the K-*ras *4A/4B splice variants is reduced in human sporadic CRC in both primary adenocarcinomas and colon cancer cell lines that harbour K-*ras *activating mutations, including homozygous mutations [11,12]. Since mutationally activated K-Ras 4B has an anti-apoptotic action [13,14] and, unlike K-Ras 4A, can promote cell migration [10], and K-Ras 4B can drive expression of matrix metalloproteinase 2 (MMP-2) which specifically cleaves type IV collagen, and is involved in cell detachment and migration [15], the altered splicing of the K-*ras *oncogene in CRC in favour of K-*ras *4B could contribute to neoplastic progression by enabling the survival of cells with DNA damage and facilitating tumour invasion and, ultimately, metastasis. Indeed, tumour growth and metastasis in human CRC is linked with increased expression of MMP-2 [see [7]]. However, the finding that the K-*ras *4A/4B ratio is also reduced in CRC cell lines that lack K-*ras *activating mutations raises the possibility that a regulated switch in alternative splicing of the K-*ras *proto-oncogene may also have a causal role in tumour progression [12]. The mechanism could involve increased expression of MMP-2 (see above), and/or reduced apoptosis given that the K-Ras 4A proto-oncoprotein exerts a pro-apoptotic action in mouse intestine following etoposide-induced DNA damage, and evidence that the K-Ras proto-oncoproteins have antagonistic effects on apoptosis in embryonic stem (ES) cells: Ras 4A promotes, whereas K-Ras 4B inhibits, apoptosis [16]. Further, while K-Ras 4B, and probably K-Ras 4A, promote ES cell differentiation following withdrawal of leukaemia inhibitory factor [16] it is unlikely they do so with similar efficiency since the Raf/MAPK pathway regulates stem cell differentiation [reviewed [17]] and K-Ras 4A and 4B differ in their ability to activate Raf-1 [10]. Therefore, in accordance with the 'stem cell model' for cancer formation we proposed that a change in the ratio of K-Ras proto-oncoproteins may further contribute to neoplastic progression by perturbing stem cell differentiation [see [12]]. Thus, altered splicing of the K-*ras *proto-oncogene could drive tumour progression in sporadic CRC by promoting MMP-2 expression, and inappropriate stem cell survival and self-renewal.

To address the hypothesis that alteration in the ratio of the K-Ras proto-oncoproteins in favour of K-Ras 4B can affect tumour formation in the small intestine in the absence of K-*ras *activating mutations, K-*ras*^tmΔ4A/tmΔ4A ^mice, which express the K-*ras *4B splice variant only [18], were crossed with *Apc*^Min/+ ^(*Min*) mice. The latter mice harbour a heterozygous germ-line nonsense mutation in the *Apc *(adenomatous polyposis coli) tumour suppressor gene, and are predisposed to developing multiple intestinal tumours initiated by loss of the wild-type *Apc *allele [reviewed [19]]. By this means tumorigenesis in the small intestine was compared between *Apc*^Min/+^, K-*ras*^+/+ ^and *Apc*^Min/+^, K-*ras*^tmΔ4A/tmΔ4A ^mice that can, and cannot, express K-*ras *4A respectively. This approach was selected since K-*ras*^tmΔ4A/tmΔ4A ^mice are healthy [16,18], intestinal tumours in *Min *mice do not harbour K-*ras *activating mutations [20], and K-Ras 4A deficiency does not affect K-*ras *4B expression in the small intestine [16] where, importantly, most (> 95%) intestinal tumours form in *Min *mice [reviewed [19]]. Thus, the effect of K-Ras 4A on tumorigenesis can be examined in the *absence *both of its oncogenic allele and of alteration in K-*ras *4B expression as a consequence of K-Ras 4A deficiency.

## Methods

### Mice

All animal work was carried out using procedures approved by the ethical panel of the University of Edinburgh and licensed by the Home Office (Project licence PPL60/3433). Inbred Apc^Min/+ ^mice (C57BL/6) were obtained from The Jackson Laboratory (Bar Harbor, Maine, USA). The K-*ras*^tmΔ4A/tmΔ4A ^mouse has been reported previously [18]. Since penetrance of the *Apc*^Min ^mutation is affected by genetic background, the K-*ras*^tmΔ4A ^allele on a F1(129/Ola × C57BL/6) background was backcrossed for a further 8 generations on to the susceptible C57BL/6 inbred background. Resultant K-*ras*^tmΔ4A/tmΔ4A ^mice were confirmed as congenic for the C57BL/6 *Pla2g2a *allele (*Mom-1*) by PCR as described previously [21]. Inbred Apc^Min/+^, K-*ras*^+/+ ^× Apc^+/+^, K-*ras*^tmΔ4A/tmΔ4A ^crosses generated *Apc*^Min/+^, K-*ras*^tmΔ4A/+ ^and *Apc*^+/+^, K-*ras*^tmΔ4A/+ ^mice. Male *Apc*^Min/+^, K-*ras*^tmΔ4A/+ ^mice were crossed with female *Apc*^+/+^, K-*ras*^tmΔ4A/+ ^mice to generate informative *Apc*^Min/+^, K-*ras*^+/+ ^and *Apc*^Min/+^, K-*ras*^tmΔ4A/tmΔ4A ^offspring. Mice heterozygous for the *Apc*^Min ^allele were identified by PCR as described previously that generates a wild-type (123 bp) and mutant *Apc*^Min ^(144 bp) band [22]. The K-*ras *genotype was determined using primers that amplify K-*ras *exon 4A, and primers that identify the mutant K-*ras*^tmΔ4A ^allele by amplifying the *neo *cassette which replaces K-*ras *exon 4A, that generate 72 bp and 206 bp bands respectively [18]. The genotyping of mice is summarised in Table 1. Survival curves for *Apc*^Min/+^, K-*ras*^+/+ ^and *Apc*^Min/+^, K-*ras*^tmΔ4A/tmΔ4A ^mice were compared using the Mantel-Haenszel test [23]. Mice were culled immediately on showing signs of intestinal neoplasia, including anaemia, pale feet, hunching and/or swollen abdomen. A small number of mice (3 male and 4 female *Apc*^Min/+^, K-*ras*^+/+ ^mice, and 4 male and 2 female *Apc*^Min/+^, K-*ras*^tmΔ4A/tmΔ4A ^mice) with rectal prolapse, which were immediately culled, and also mice randomly withdrawn for tumour counts were treated as censored observations [23]. Reclassification of mice with prolapse as uncensored observations did not affect any of the conclusions of the study.

**Table 1:** Genotyping litters from *Apc*^Min/+^, K-*ras*^tmΔ4A/+ ^× *Apc*^+/+^, K-*ras*^tmΔ4A/+ ^crosses to identify informative *Apc*^Min/+^, K-*ras*^+/+ ^and *Apc*^Min/+^, K-*ras*^tmΔ4A/tmΔ4A ^offspring

**Genotype**	***Apc***	***neo***	**K-*ras *exon 4A**
*Apc*^Min/+^, K-*ras*^+/+^	+ (123 bp, 144 bp)	-	+ (72 bp)
*Apc*^Min/+^, K-*ras*^tmΔ4A/+^	+ (123 bp, 144 bp)	+ (206 bp)	+ (72 bp)
*Apc*^Min/+^, K-*ras*^tmΔ4A/tmΔ4A^	+ (123 bp, 144 bp)	+ (206 bp)	-
*Apc*^+/+^, K-*ras*^+/+^	+ (123 bp)	-	+ (72 bp)
*Apc*^+/+^, K-*ras*^tmΔ4A/+^	+ (123 bp)	+ (206 bp)	+ (72 bp)
*Apc*^+/+^, K-*ras*^tmΔ4A/tmΔ4A^	+ (123 bp)	+ (206 bp)	-

+/- = PCR product present/absent respectively (see Methods)

### Histology

Mice were killed by CO_2 _asphyxiation and the small intestine removed, flushed with phosphate buffered saline, opened lengthwise, and mounted *en face*. Following fixation for 4 hours in methacarn (4 volumes methanol, 2 volumes chloroform and 1 volume glacial acetic acid) the number and area of all visible tumours was scored using a dissection microscope at 10× magnification.

For examination of tumour pathology, "gut roll" preparations were made from mice with overt signs of neoplasia as described above, fixed in 10% buffered formalin, wax embedded, and stepped serial sections stained with haematoxylin and eosin. Mitotic and apoptotic counts were defined as the mean number of mitotic or apoptotic figures in a single high-power (× 400) microscope field within each of ten adenomas of the small intestine of comparable size and dysplasia in preparations from five or six individual mice (of both sexes combined) of each genotype, counting only intra-epithelial figures and avoiding debris in glandular lumina and any non-neoplastic epithelium on the adenoma surface.

Tumour number, area, and mitotic and apoptotic counts were compared by Student's *t*-test.

### Analysis of K-*ras* mutations by PCR-direct sequencing

DNA was prepared from individual tumours dissected from the small intestine and screened for the presence of K-*ras *mutations at codons 12, 13 and 61 as described previously [20].

### Analysis of K-*ras* 4A and K-*ras* 4B transcript expression levels by quantitative RT-PCR

Normal small intestinal tissue samples and tumours (at least 2 mm diameter, from mice with overt signs of neoplasia as described above, care being taken to avoid Peyer's patches) were dissected and frozen in "RNA later" buffer (Sigma) for subsequent RNA extraction. Total RNA (100 ng) was reverse transcribed in 25 μl volume using the iTaq SYBR Green RT-PCR kit (Bio-Rad) following the manufacturer's instructions. All real-time quantitative reverse transcription polymerase chain reactions (RT-qPCR) were amplified starting with denaturation at 95°C for 3 min, then 45 cycles of 95°C for 15 sec and 60°C for 1 min. The following exon-spanning primers were used: mouse *β-actin *upstream primer (5'-AAGCTGTGCTATGTTGCTCTAGACT-3'), and downstream primer (5'-CACTTCATGATGGAATTGAATGTAG-3'); mouse K-*ras *4A upstream primer (5'-CCTGGTAGGGAATAAGTGTGATTTG-3'), and downstream primer (5'-GTACTGTCGGATCTCTCTCACCAAT-3'); mouse K-*ras *4B upstream primer (5'-GAGTAAAGGACTCTGAAGATGTGCC-3') located in K-*ras *exon 3, and downstream primer (5'-CATCGTCAACACCCTGTCTTGTCTT-3') that spans the junction of K-*ras *exon 3 and 4B (specific for the mouse K-*ras *4B transcript). The PCR product sizes derived from K-*ras *4A, K-*ras *4B and *β-actin *transcripts were 185 bp, 158 bp and 148 bp respectively. The specificities of the PCR reactions were confirmed by dissociation curve analysis and 2% agarose gel electrophoresis. All PCR products were analysed when in the exponential phase of PCR amplification. Quantification of the relative expression levels of K-*ras *4A and K-*ras *4B transcripts was performed using standard curves with normalization against those of *β-actin *transcripts from the same sample. The relative values were corrected for dilution factors and then corrected for the differences in size of 4A and 4B amplified products. Because the DNA binding affinities of the PCR primers and the sizes of the amplified products were closely similar for the K-*ras *4A and K-*ras *4B PCR reactions, and the standard curves also showed that the PCR efficiencies for the K-*ras *4A and K-*ras *4B PCR reactions were very similar at 74.8% and 76.1% respectively, the expression levels of these two different transcripts, K-*ras *4A and K-*ras *4B, can be compared relative to each other, setting the level of expression of K-*ras *4A in wild-type C57BL/6 mouse lung as an arbitrary value of 1.0.

## Results

**Figure 1:**
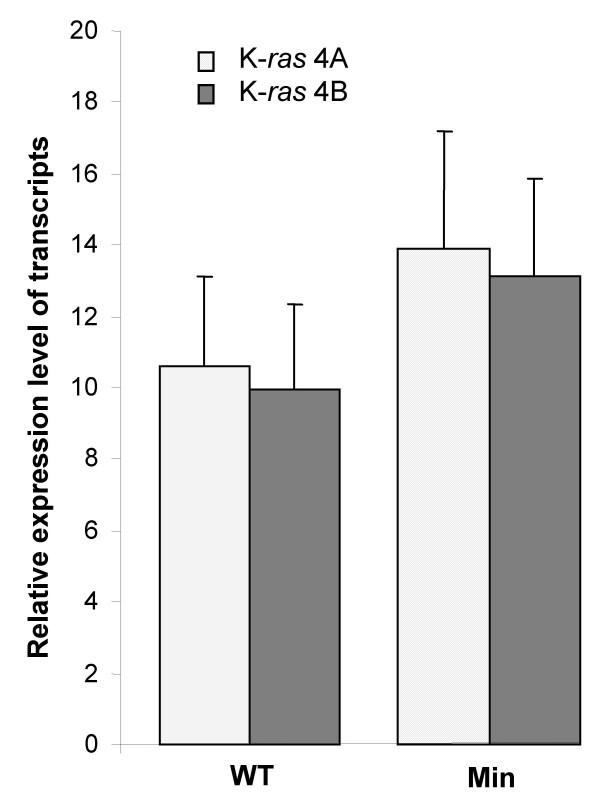
**Real-time RT-qPCR analysis showing relative expression of the K-*ras *4A and 4B transcripts (mean ± SD) in small intestine (duodenum) from 8 week-old, inbred (C57BL/6) wild-type (WT) and *Apc*^Min/+ ^(*Min*) mice (6 male mice were analysed in each cohort).** Light (stippled) bars, K-*ras *4A; dark (hatched) bars, K-*ras *4B.

Both K-*ras *splice variants are expressed in mouse small intestine [18,24]. In the present study the relative levels of the K-*ras *4A and 4B transcripts were quantified by real-time RT-qPCR analysis. *Min *mice were examined at 8 weeks-old before they showed any visible sign of intestinal tumorigenesis and, for control purposes, comparisons were made with age-matched wild-type mice. The K-*ras *4A/4B transcript ratio did not differ significantly (*P *= 0.95) between wild-type (1.07 ± 0.09) and *Min *(1.07 ± 0.15) mice, and in both cases the K-*ras *4A transcript accounted for about 50% of K-*ras *expressed in the small intestine (Figure 1).

**Figure 2:**
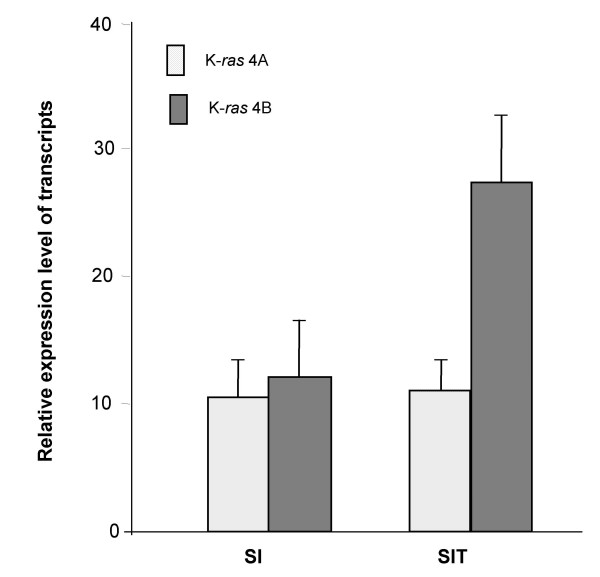
**Real-time RT-qPCR analysis showing relative expression of the K-*ras *4A and 4B transcripts (mean ± SD of 6 samples in each case) in normal small intestine (SI) and tumours of the small intestine (SIT) of *Apc*^Min/+ ^mice with overt signs of neoplasia.** Light (stippled) bars, K-*ras *4A; dark (hatched) bars, K-*ras *4B.

**Figure 3:**
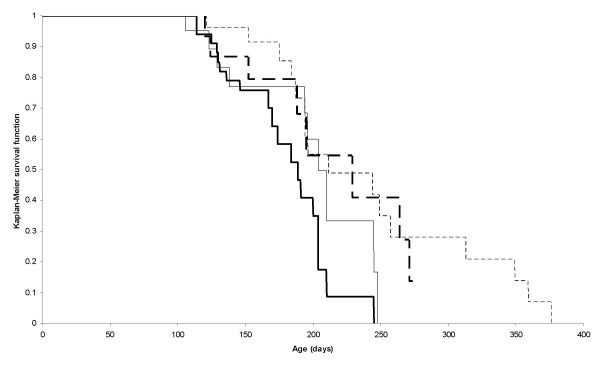
**Survival curve showing *Apc*^Min/+^, K-*ras*^+/+ ^mice [*n *= 20 males (heavy continuous line), *n *= 8 females (heavy broken line)] and *Apc*^Min/+^, K-*ras*^tmΔ4A/tmΔ4A ^mice [*n *= 10 males (light continuous line), *n *= 16 females (light broken line)].** In each case *n *refers to the number of uncensored observations.

The reduced K-*ras *4A/4B splice variant ratio in sporadic human CRC involves down-regulation of K-*ras *4A expression [11]. In *Min *mice (Figure 2), while there was no significant difference between K-*ras *4A and 4B transcript levels in normal small intestine (*P *= 0.383), tumours of the small intestine showed significantly reduced levels of K-*ras *4A relative to K-*ras *4B transcripts (*P *= 0.0015). However, the reduced K-*ras *4A/4B splice ratio was due to an increase in the level of K-*ras *4B transcript expression, while the level of K-*ras *4A transcripts was not appreciably altered. While this provides no support for the hypothesis that K-*ras *4A plays a role in modulating intestinal tumorigenesis, it does not exclude this hypothesis, which is worthy of further examination given the high expression levels and established pro-apoptotic action of K-*ras *4A in mouse intestine and the antagonistic effects of the 4A and 4B isoforms [16]. Therefore, to determine if the K-Ras 4A proto-oncoprotein can modulate tumorigenesis in the small intestine comparisons were made between age-matched *Apc*^Min/+^, K-*ras*^+/+ ^and *Apc*^Min/+^, K-*ras*^tmΔ4A/tmΔ4A ^mice. Since gender may influence tumour development in the *Min *mouse [see [25]] males and females were considered separately. Mice were sampled randomly from the stock at 106 days when the stock was outwardly healthy, and at 152 days when 17% (19/112) of the stock had been either found dead or necessitated necropsy due to sickness [20% (12/59)*Apc*^Min/+^, K-*ras*^+/+ ^and 13% (7/53) *Apc*^Min/+^, K-*ras*^tmΔ4A/tmΔ4A^]. Examination of the small intestine revealed a similar overall tumour number, and no difference in tumour size for both male and female mice at either time point (Table 2). Histological analysis of intestinal preparations from *Apc*^Min/+^, K-*ras*^+/+ ^(*n *= 8) and *Apc*^Min/+^, K-*ras*^tmΔ4A/tmΔ4A ^(*n *= 7) mice with overt signs of neoplasia revealed that all tumours from both genotypes were adenomas of similar, mild to moderate, dysplasia, with no evidence of invasive carcinoma in any of them. There was no statistically significant difference between the genotypes in mitotic counts (*Apc*^Min/+^, K-*ras*^+/+^: 25.3; *Apc*^Min/+^, K-*ras*^tmΔ4A/tmΔ4A^: 26.8; *P *= 0.468) or apoptotic counts (both genotypes: 2.1; *P *= 1.0) in the tumours. Consistent with the tumour data *Apc*^Min/+^, K-*ras*^+/+ ^and *Apc*^Min/+^, K-*ras*^tmΔ4A/tmΔ4A ^mice exhibited similar survival (Figure 3) for both males (χ^2^_1 _= 1.91, P = 0.167) and females (χ^2^_1 _= 0.44, P = 0.506).

**Table 2:** Tumorigenesis in the small intestine of outwardly healthy *Apc*^Min/+^, K-*ras*^+/+ ^and *Apc*^Min/+^, K-*ras*^tmΔ4A/tmΔ4A ^mice

**Genotype**	**Sex**	**Mice per cohort**	**Age (days) **± SEM	**Tumour number **± SEM	**Tumour area (mm^2^) **± SEM
**Stocks culled at ≅ 106 days**					

*Apc*^Min/+^, K-*ras*^+/+^	M	4	108.3 ± 1.0	36.3 ± 7.9^*a*^	43.5 ± 9.4^*i*^
*Apc*^Min/+^, K-*ras*^+/+^	F	4	104.5 ± 1.5	59.0 ± 2.9^*b*^	74.3 ± 8.8^*j*^
*Apc*^Min/+^, K-*ras*^tmΔ4A/tmΔ4A^	M	6	107.2 ± 0.5	51.5 ± 16.2^*c*^	68.8 ± 23.1^*k*^
*Apc*^Min/+^, K-*ras*^tmΔ4A/tmΔ4A^	F	4	105.5 ± 0.5	36.5 ± 15.3^*d*^	57.0 ± 29.8^*l*^

**Stocks culled at ≅ 152 days**					

*Apc*^Min/+^, K-*ras*^+/+^	M	10	152.0 ± 0.9	56.8 ± 8.8^*e*^	131.0 ± 19.6^*m*^
*Apc*^Min/+^, K-*ras*^+/+^	F	4	151.8 ± 1.3	46.3 ± 5.9^*f*^	89.8 ± 10.9^*n*^
*Apc*^Min/+^, K-*ras*^tmΔ4A/tmΔ4A^	M	3	153.3 ± 1.3	35.3 ± 7.5^*g*^	99.0 ± 20.0^*o*^
*Apc*^Min/+^, K-*ras*^tmΔ4A/tmΔ4A^	F	9	151.1 ± 2.8	61.3 ± 9.7^*h*^	118.7 ± 22.3^*p*^

M, male; F, female. ^*a *^*vs.*^*c *^*P *= 0.42;^*b *^*vs.*^*d *^*P *= 0.20; ^*e *^*vs.*^*g *^*P *= 0.090;^*f *^*vs.*^*h *^*P *= 0.21; ^*i *^*vs.*^*k *^*P *= 0.34;

^*j *^*vs.*^*l *^*P *= 0.60; ^*m *^*vs.*^*o *^*P *= 0.28; ^*n *^*vs.*^*p *^*P *= 0.27 (Student's *t*-test in each case).

Previous studies have established that intestinal tumours from *Apc *mutant mice, including adenocarcinomas, do not harbour K-*ras *activating mutations [20,26,27]. In confirmation, and to establish this applies in compound mutant *Min *mice, K-*ras *codons 12, 13 and 61 were sequenced in 27 small intestinal tumours, that included 8 tumours from *Apc*^Min/+^, K-*ras*^+/+ ^mice, 15 tumours from *Apc*^Min/+^, K-*ras*^tmΔ4A/+ ^mice, and 4 tumours from *Apc*^Min/+^, K-*ras*^tmΔ4A/tmΔ4A ^mice. No tumours were found to harbour a K-*ras *activating mutation (data not shown).

## Discussion

Because intestinal tumours develop spontaneously in *Apc *mutant mice they present a powerful model system to study factors that modulate intestinal tumour development, including genetic modifiers, which may be important in the design of potential therapeutic strategies [reviewed [28]]. To date, inactivating mutations in genes involved in arachidonic acid and sphingosine metabolism, DNA methylation, and genes encoding the matrix mellalloproteinase MMP-7, VEGF-A and matricellular proteins have been found to suppress intestinal tumorigenesis in *Apc *mutant mice. Conversely, inactivating mutations in genes related to genomic stability and DNA mismatch repair, and genes that regulate PI3-K/AKT signalling, and proliferation and differentiation in the crypt-villus axis, promote tumorigenesis [see [19,25,28-38]].

Because homozygosity for the K-*ras *null allele is embryonic lethal [39] nothing is known about the effect of inactivation of the K-*ras *proto-oncogene on intestinal homeostasis. The present study found the K-Ras 4A proto-oncoprotein, whose transcript accounts for some 50% of K-*ras *expressed in the small intestine, does not affect tumour development in the *Min *mouse intestine where tumours lack K-*ras *activating mutations. The result is perhaps unexpected given that the K-*ras *proto-oncogene can promote ES cell differentiation, exert a pro-apoptotic action in ES cells following DNA damage induced by etoposide or cisplatin [16,40,41] and, importantly, can suppress tumour development in the *absence *[41] as well as the presence [42] of its oncogenic allele. Moreover, the K-Ras 4A proto-oncoprotein exerts a pro-apoptotic action in both ES cells and mouse small intestine following etoposide-induced DNA damage [16]. The failure of K-Ras 4A to affect tumorigenesis may reflect the fact that K-Ras 4A deficiency does not affect baseline levels of apoptosis in the crypt [16] and/or abolish completely the apoptotic response in the small intestine following DNA damage, but rather it is reduced and delayed, which implies activation of K-Ras 4A-independent apoptotic pathways. The complex relationship between apoptosis and cancer development is highlighted by recent evidence that the tumour suppressor activity of *p53 *in radiation-induced lymphoma is not dependent on its ability to promote apoptosis and so eliminate mutant cells [43]. Therefore, the K-Ras 4A-mediated pro-apoptotic response in the small intestine following etoposide-induced DNA damage could be irrelevant for tumour formation and, indeed, it does not necessarily follow that K-Ras 4A has a pro-apoptotic action at physiological levels of DNA damage, or with other types of DNA damage. Thus, while the pattern of K-*ras *4A expression is strongly conserved in human and mouse tissues [12,18] its role in intestinal homeostasis remains unclear. However, given that K-Ras 4B can promote ES cell differentiation and suppress mammary carcinogenesis in the *absence *of its oncogenic allele [16,44] it remains to be determined whether K-Ras 4B can suppress intestinal tumorigenesis. This would necessitate a comparison between *Apc*^Min/+ ^mice that can, and cannot, express K-*ras *4B. However, since K-*ras *is essential for mouse development [39] and K-*ras *4B is the major splice variant and, unlike K-*ras *4A, is expressed ubiquitously [12,18], it is doubtful that K-Ras 4B-deficient mice would be viable, and therefore such a study would require conditional inactivation.

In sporadic CRC the K-*ras *4A/4B splice variant ratio is reduced in colon cancer cell lines, regardless of whether they harbour K-*ras *activating mutations [11,12]. Since the K-*ras *4A/4B ratio is reduced in primary CRC tumours [12] the altered ratio in cell lines, including those that lack K-*ras *activating mutations, is more likely to be symptomatic of the tumours from which they were derived rather than an *in vitro *artefact of the growth conditions. While altered splicing of the K-*ras *proto-oncogene in favour of K-*ras *4B could, conceivably, modulate tumour progression, and possibly by effects on MMP-2 expression, apoptosis and/or differentiation (see Background), the present study found that alteration in the ratio of the K-Ras proto-oncoproteins in favour of K-Ras 4B (by targeted deletion of K-*ras *exon 4A) does not affect mouse survival or tumour number, size or histopathology (including mitotic and apoptotic counts). These observations are unlikely to reflect the fact that *Min *mice die before intestinal tumours can progress, since adenocarcinomas do develop in compound mutant *Min *mice [see [19,29-31,36]]. They could relate to the fact that the altered ratio in human CRC involves not only an decrease in K-*ras *4A expression but also an increase in K-*ras *4B expression [11]: indeed, in *Min *mouse tumours, K-*ras *4B expression is elevated while K-*ras *4A expression is not appreciably altered. K-*ras *4B expression was unaffected in the comparisons shown in Table 2 and Figure 3 because K-*ras *4A deficiency does not affect K-*ras *4B expression in the small intestine [16]. Given that K-*ras *4B, unlike K-*ras *4A, can promote cell migration and MMP2 expression [10,15], the possibility that increased expression of K-*ras *4B, with or without reduced expression of K-*ras *4A, is an essential component for tumour progression in cases that lack K-*ras *activating mutations remains to be addressed. Although the present study establishes that a reduction in the K-*ras *4A/4B ratio does not affect *Apc*-driven intestinal tumorigenesis *per se *the finding that the ratio is reduced in *Min *adenomas that lack K-*ras *activating mutations raises the intriguing possibility that K-*ras *may have a more widespread role in tumorigenesis in addition to that in lung, colon and pancreatic cancers that normally harbour K-*ras *activating mutations [reviewed [6]]. Thus, given that K-*ras *4A and 4B are co-expressed widely in mammalian tissues [12,18] it remains to be determined whether their ratio is altered in other types of tumours that routinely lack K-*ras *activating mutations.

## Conclusion

Even though the K-Ras 4A proto-oncoprotein exerts a pro-apoptotic action in the small intestine following etoposide-induced DNA damage, it does not affect tumour development, albeit when expressed in the *absence *of its oncogenic allele.

## Competing interests

The authors declare that they have no competing interests.

## Authors' contributions

CEP, study design, performed experimental studies, drafted the manuscript; MJA & FL, real-time RT-qPCR analysis, histopathology, edited the manuscript; LR, MW, PSD, RLB, NJL & RAR, mouse breeding, genotyping and tumour studies; OJS, contribution to study design, provision of cDNAs, edited the manuscript; MLH, statistical analysis, edited the manuscript. All authors read and approved the final manuscript.

## Pre-publication history

The pre-publication history for this paper can be accessed here:


